# The official websites of blood centers in China: A nationwide cross-sectional study

**DOI:** 10.1371/journal.pone.0182748

**Published:** 2017-08-09

**Authors:** Huiying Hu, Jing Wang, Ming Zhu

**Affiliations:** Department of Blood Resource Management, Institute of Blood Transfusion, Chinese Academy of Medical Sciences & Peking Union Medical College, Chengdu, PR. China; FDA, UNITED STATES

## Abstract

**Background:**

Blood collection agencies worldwide are facing ongoing and increasing medical demands for blood products. Many potential donors would search related information online before making decision of whether or not to donate blood. However, there is little knowledge of the online information and services provided by blood centers in China, despite the constantly increase of internet users. Our research investigates the number of blood centers’ official websites and their quality, and highlights the deficiencies that required future advances.

**Methods:**

Identified official websites of blood centers were scored using a newly developed evaluation instrument with 42 items concerning technical aspects, information quality, information comprehensiveness and interactive services. Scores of websites were compared between blood centers with different level (provincial vs. regional blood centers) and location (blood centers located in economically developed vs. developing region).

**Results:**

For the 253 working official websites all the 350 blood centers in China, and the mean overall score of websites was 24.7 out of 42. 79.1% websites were rated as fair (50–75% of maximum), 5.5% as good (≥75% of maximum) and 15.4% as poor(25–50% of maximum;). Websites got very low sub-scores in information quality (mean = 3.8; range 1–8; maximum = 9) and interactive services (3.3; 0–10; 10). Higher proportions of provincial (vs. regional) blood centers and economically developed (vs. developing) blood centers had official websites (p = 0.044 and p = 0.001; respectively) with better overall quality (p<0.001 and p <0.01) and better sub-scores (in all of the four sections and in technical aspects and information quality). Website overall scores was positively correlated with the number of people served by each blood center (p< 0.001) and the donation rate of each province (p = 0.046).

**Conclusions:**

This study suggests there is a need to further develop and improve official websites in China, especially for regional and inland blood centers. The poor information quality and interactive services provided by these websites is of particular concern, given the challenges in blood donor counselling and recruitment.

## Introduction

The medical demands for blood supplies worldwide are increasing, which lead to an immense need to ensure safe and sufficient supply of blood products. In China, the increase in the blood supply has not kept pace with the increasing clinical demand for blood [1] and only a small volunteering group of people (around 0.9% of the total population) have donated blood. Recruiting and retaining blood donors are the key challenges for blood centers [2].

Knowledge, attitude and practice surveys on donors and potential donors showed that many eligible residents do not have enough knowledge or correct understanding about blood donation, especially in developing countries [3] like China [4]. Conventional ways of providing information and education, including paper-based written brochure and video approaches have been shown to enhance donation attitudes and intentions to give blood. However, these educational materials are often provided to donors at the time of recruitment, around the blood collection site or send to specific group of people [5, 6]. Accessible anywhere with connection to internet at any time, online donation information and services may offer superior convenience to potential donors. A blood center in the Los Angeles metropolitan area arranges approximately 25% of donations by their customized website [7]. An interactive web site addressing common fears and concerns about donating had higher efficacy in enhancing participants’ donation attitudes, reducing donation anxiety and boosting intentions to donate blood [8]. From the users’ perspective, a survey showed that prospective donors wanted to be informed about organizational details of blood donation such as opening times and laboratory tests performed [9], and official internet campaign was pointed out to be an effective way for endorsing the idea of giving blood by another interview [10].

According to the 38th Statistical Report on Internet Development in China, as of June 2016, there were 710 million people in China with access to the Internet [11]. The Internet has become a part of daily life for many young people, who are the main age group of voluntary blood donors in China [12]. As a result, qualified websites would influences these users and potentially promote blood donation. Although a few official websites of blood centers have been reported [13], little is known about the whole profile of blood centers’ websites. The aim of this study was to explore the number and quality of blood centers’ official websites in China and define drawbacks to promote targeted improvements of these websites.

## Materials and methods

### Website identification

According to the latest national survey on blood supply (conducted in August, 2015), all blood centers in mainland China are state-owned public organizations divided into three levels: provincial, regional and county. Provincial blood centers (n = 32) and regional blood centers (n = 318) are located in the provincial capitals and second-tier (smaller) cities, while county blood centers are gradually being turned into branches of regional blood centers [14]. Therefore, only official websites of provincial and regional blood centers were included for evaluation.

Based on the full list of provincial and regional blood centers [14], two authors (HH and JW) independently searched the term “the name of city/ province + blood center/ bank” both in English and Chinese by using country specific version of Google for China (www.google.hk.com) and a common search engine Baidu (www.baidu.com) on September 10, 2016. Then the identified websites were examined by the third author (MZ) and any inconsistency or conflict was resolved by discussion. The following criteria were applied to determine if the website was the working official websites of a provincial/ regional blood center: 1) was a standard website, but not a single page, discussion forum or blog; 2) provided valid Internet Content Provider (ICP) serial number and a public institution Certificate of Identity (given and verified by the China Organization Name Administration Center); 3) had been regular updated (evaluated by the latest date on the website within one year before the time of review).

### Development and validation of the evaluation instrument

Comprehensive literature review revealed that there was no specific website evaluation instrument for blood centers. Since the services provided by blood centers are comparable to hospitals or clinics in some aspects, our evaluation framework was developed based on evaluation instruments for websites of hospitals [15] and obstetrics departments [16]. Validated quality assessment tools for health websites and standards for online health-related information such as DISCERN instrument [17], Health on the Net Foundation (HON) code[18] and Silberg criteria [19] were used to form the evaluation sections and items. The essential content in the “Blood Donor Counselling Implementation Guideline” [20] published by WHO was used to examine the coverage of information and services. Findings from studies on website of blood centers were also included [7–10]. The preliminary instrument was applied to a sample of 10 randomly selected websites by one rater (HH). Based on the results of evaluation, item modification was carried out after discussion.

The final evaluation instrument consisted of 42 items in four sections: (1) technical aspects. Ten items assessed the technical implementation details of the websites. (2) Information quality. Nine items evaluated the quality of information, such as the authority, attribution and currency of information. (3) Information comprehensiveness. Thirteen items assessed whether the scope of information and the level of detail provided were adequate for donor counselling. (4) Interactive services: Ten items represent the communicational and transactional services which are suitable to be provided online, including on-line consultation, donation appointment and so on. Raters independently searched the direct page and all other pages through navigation to undertake evaluation, and the evaluation score were defined as 1 for specific content, component or functionality found and 0 for not found. Sub-score was the sum of scores of items in each of the four sections. Items were weighted equally in the four sections, and the overall score is the sum of all the four sub-scores.

To ensure the reliability of the instrument, a preliminary study was conducted. A random sample of identified official websites (20% of the total websites, n = 50) was selected. Two raters (HH and WJ) each rated 25 randomly selected websites independently and discussed any discrepancies in the decisions made. The third rater (ZM) reexamined these 50 websites and the concordance among raters was analyzed by dividing the number of agreed observations by the total observations (94.79%). In the formal evaluation process, three raters independently rated a sample of randomly assigned websites (n = 84, 84 and 85; respectively).

### Statistical analysis

Websites overall scores and sub-scores were calculated and compared between provincial vs. regional blood centers, given the administrative structure of blood centers in China [2]. Provincial blood centers and regional blood centers are located in the provincial capitals and smaller cities in each province, with the main responsibility of collecting and supplying blood. The former one also should have the capability of providing quality evaluation, business instruction and staff training for blood centers in the same province [21]. We used the gross domestic product (GDP) per capita of each province in 2015 [22] to capture the apparent regional inequality of economic and social development in China [23], and compared the website overall scores and sub-scores between blood centers located in economically developed vs. developing regions. The economically developed regions include ten provinces with the highest GDP per capita (all > $1,000): Tianjin, Beijing, Shanghai, Jiangsu, Zhejiang, Inner Mongolia, Fujian, Guangdong Liaoning and Shandong. The other provinces were classified as the developing regions, including Hebei, Shanxi, Guangxi, Henan, Anhui, Hubei, Hunan, Jiangxi, Shaanxi, Ningxia, Gansu, Sichuan, Guizhou, Yunnan, Qinghai, Tibet and Xinjiang (S1 Fig and in S1 Table). The chi-square test and the Fisher exact test (if expected cell counts were<5) were used for comparing categorical variables and the Mann–Whitney U test was used to compare overall scores and sub-scores with a significance value of 0.05.

To see if the website evaluation scores are associated with the working capacity of blood centers, we collected data on the number of people served by each blood center (S2 Table) and Spearman correlation coefficients and regression coefficients were calculated. Only the data about blood donation of each province was available, so the website overall score of blood centers in each province were averaged to get a provincial mean score (S3 Table) and the correlation between provincial mean score and blood donation data were examined. All the analyses were conducted using R statistical software (R Development Core Team, 2015).

## Results

### Working websites of blood centers

**Fig 1:**
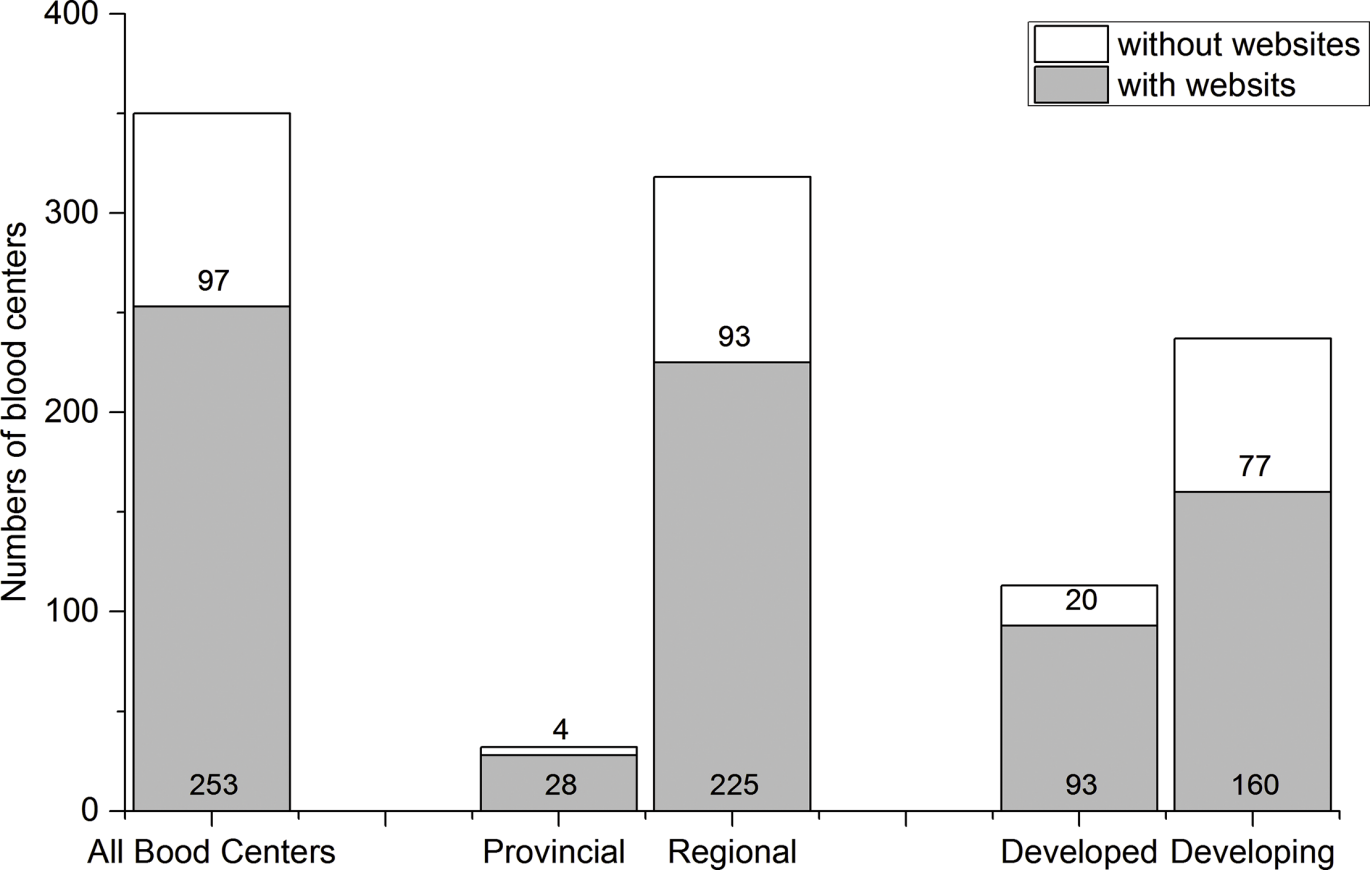
The numbers of blood center with or without a working official website. Numbers within stacked bars show the number of websites within each category.

Among 350 provincial/ regional blood centers, 253 blood centers (72.3%) had a working official website. The percentage of provincial blood centers with a website was significantly higher than regional ones (87.5% vs. 70.8%; p = 0.044), and the economically developed blood centers had significantly more working official websites than developing ones (82.3% vs. 67.5%; p = 0.001) (Fig 1).

### Evaluation scores of websites

The overall scores and the four sub-scores achieved by all websites and websites broken down by level and location were summarized in Table 1. Although the highest overall score was 38 out of 42 (90.5% of the maximal achievable score), the mean score of all websites was just 24.7(only reached 58.8% of maximum). Provincial blood centers’ websites had significantly higher overall scores (p<0.001) and sub-scores than regional ones. Compared to economically developing blood centers, developed blood centers’ websites also achieved significantly higher overall scores (p<0.01) and sub-scores in technical aspects and information quality.

**Table 1:** Mean overall website scores and mean sub-scores, by category of blood center.

	All websites	Administrative level		Economic development level	
		Provincial	Regional	Developed	Developing
**Overall** **score (42)**	24.7(12–38)	29.4(17–38) ***	24.1(12–34)	25.9(13–38)**	24.0(12–34)
**Technical aspects(10)**	7.0(5–10)	7.4(6–10)*	6.9(5–9)	7.1(5–10)*	6.9(5–8)
**Information quality (9)**	3.8(1–8)	4.4(3–8)*	3.7(1–7)	4.2(1–8)***	3.5(1–7)
**Information comprehensiveness(13)**	10.7(2–13)	11.4(7–13)*	10.6(2–13)	10.8(2–13)	10.6(3–13)
**Interactive services(10)**	3.3(0–10)	6.2(0–10)***	2.9(0–10)	3.8(0–10)	3.0(0–8)

Overall scores and sub-scores for all websites and subcategories of websites were given as mean (range). Levels of statistical significance for differences between provincial vs. regional and economically developed vs. developing blood centers blood centers are marked with: *** (p<0.001, ** (p<0.01), or *(p<0.05).

**Fig 2:**
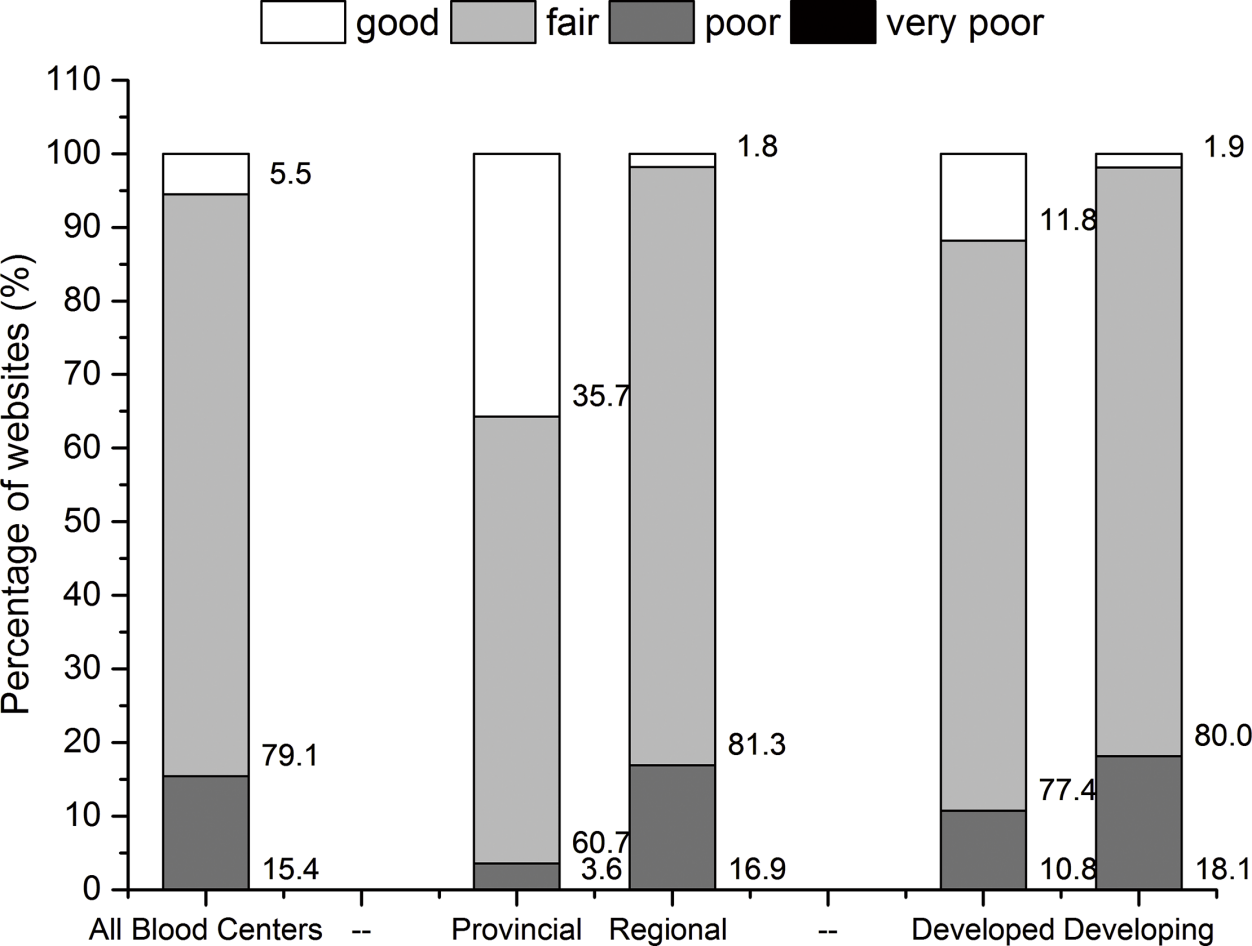
The classification of websites based on overall scores. The stacked bars show the percentages of websites classified as good, fair, poor or very poor based on their overall scores (scores≧75%, ≧50%, ≧25%, <25% of the maximum scores).

Based on the overall score, each website was ranked as good (reaching at least 75% of the maximal achievable score), fair (at least 50%), poor (less than 50%) or very poor (less than 25%). Fig 2 presents the proportions of the four ranks of websites. Websites with fair quality made up to the largest proportion (79.1% of all websites and 60.7%, 81.3%, 77.4% and 80.0% in provincial, regional, economically developed and developing blood centers’ websites). 5.5% websites were rated as good while 15.4% as poor. No website was classified as very poor. Provincial blood centers had significantly more good quality websites and less poor quality websites than regional ones (p<0.001). The same significant differences also existed between economically developed and developing blood centers (p = 0.002).

**Fig 3:**
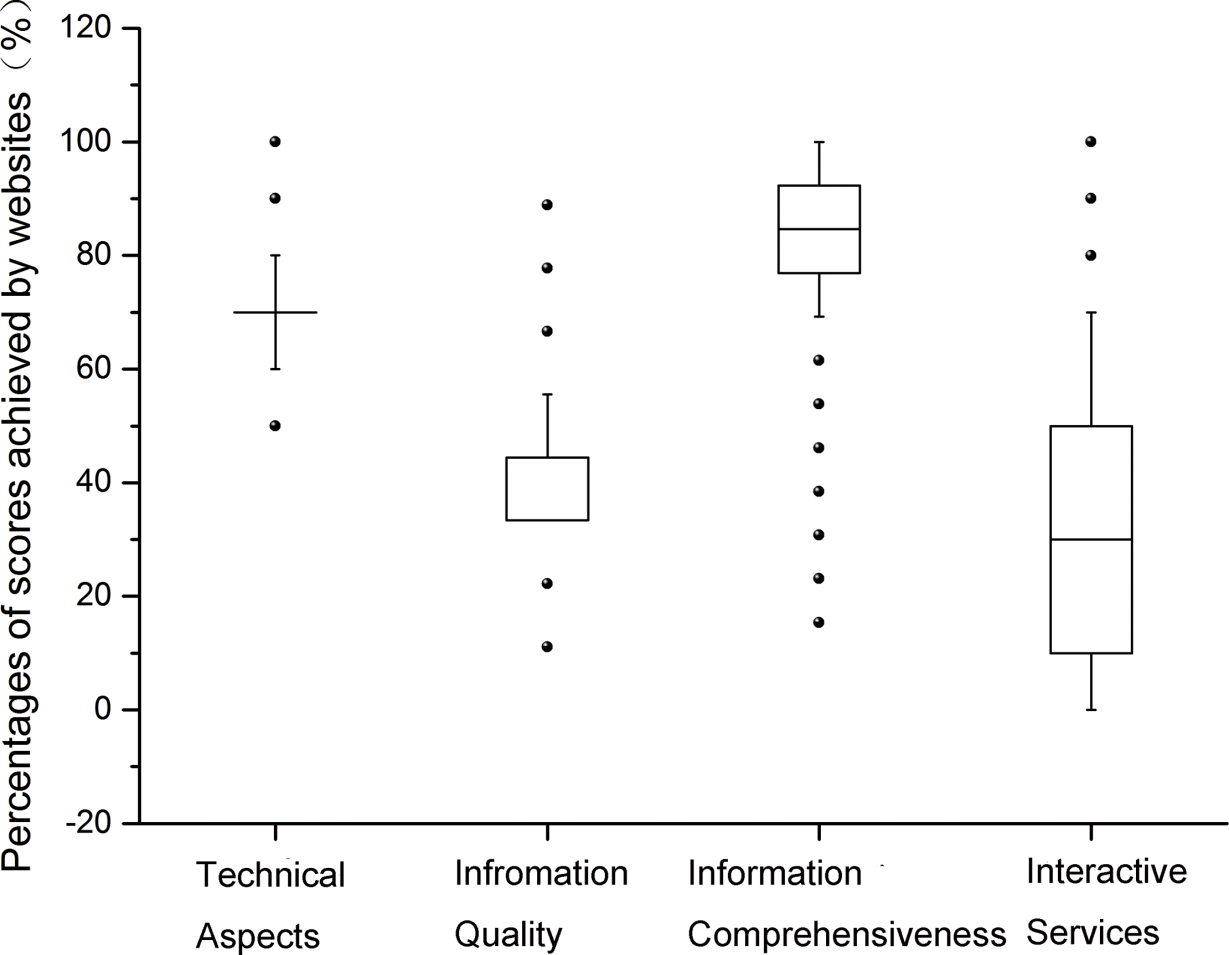
Percentages of sub-scores achieved in each section by all websites. The boundary of the box closet to zero indicates the 25^th^ percentile, the line within the box marks the median, and the boundary of the box farthest from zero indicates the 75^th^ percentile. Whiskers above and below the box indicate the 90^th^ and 10^th^ percentiles; points represent outliers.

As the four evaluation sections contained different numbers of items, percentages of sub-scores achieved were therefore calculated to compare performance of websites in each section (Fig 3). In general, websites can be regarded as fair-good in technical aspects 69.7(50.0, 100.0]) (mean, range) and information comprehensiveness 87.9(53.9, 100.0), but poor in terms of information quality (41.5 [11.1, 88.9]) and interactive services (33.5 [0.0, 100.0]). Of noted, percentages of sub-scores achieved in interactive services had a much wider range of variation than the other three sections.

### Descriptive analysis of items

Table 2 gives the descriptive analysis of each item. For all blood centers and blood centers broken down by level and location, the percentages of websites containing each item were calculated, compared and presented.

**Table 2:** Percentage of websites presenting the specific item (%).

Evaluation items	All Websites	Administrative level		Economic development level	
		Provincial	Regional	Developed	Developing
**Technical items (10)**					
1.Website map available	5.5	25.0***	3.1	8.6	3.8
2.A usable searcher engine	26.5	32.1	25.8	30.1	24.4
3. Every page includes a functional way to return to the homepage	98.8	96.4	99.1	98.9	98.8
4.Clickable links and buttons describe the related content	99.6	100.0	99.6	98.9	100.0
5. Clear content categories	100.0	100.0	100.0	100.0	100.0
6.Major headings, subheadings and main body are easily identifiable	100.0	100.0	100.0	100.0	100.0
7. Images and other multimedia relevant to, and supportive of the text content	92.5	100.0	91.6	93.5	91.9
8.Provide usable information even when JavaScript, Flash and images are disable or missing	84.2	71.4	85.8	83.9	84.4
9. Active links to useful websites (other blood centers, scientific associations, et al.)	88.1	100.0	86.7	96.8**	83.1
10.Offer information in one or more foreign languages	1.6	10.7**	0.4	3.2	0.6
**Information quality (9)**					
11. Author’s name and qualification	18.6	50.0***	14.7	26.9*	13.8
12. Mission statement	96.4	100.0	96.0	95.7	96.9
13. Privacy policies	3.2	17.9**	1.3	7.5**	0.6
14. Sources and publish date of medical information	13.4	28.6 *	11.6	20.4*	9.4
15. Contact information of webmaster	33.2	50.0	31.1	33.3	33.1
16. Financial disclosure and advertising policy	16.6	10.7	17.3	22.6	13.1
17. Copyrights notice	85.0	64.3 **	87.6	91.4	83.8
18. Disclaimers	11.9	17.9	11.1	22.6***	5.6
19. The last update time	94.9	78.6**	96.9	96.8	95.6
**Information comprehensiveness (13)**					
20. Overview of the blood center	98.4	100.0	98.2	97.8	98.8
21. Staff and Organization chart	89.3	96.4	88.4	91.4	88.1
22. Contact details of blood center	99.2	100.0	99.1	98.9	99.4
23. Knowledge on blood, blood type, transfusion and blood donation	95.7	100.0	95.1	93.6	96.9
24. Rights and responsibility of blood donors	70.0	85.7*	68.0	66.7	71.9
25. Health requirement for blood donation	81.8	82.1	81.8	79.6	83.1
26. Possible adverse events during blood donation	47.4	67.9*	44.9	55.9*	42.5
27. Explanation of registry form	88.9	96.4	88.0	89.2	88.8
28. Pre-donation health evaluation form	46.3	50.0	45.8	52.7	42.5
29. Location and guide of donation site	93.3	100.0	92.4	93.6	93.1
30. Working time for donation site	88.5	100.0*	87.1	89.2	88.1
31. Guide for reimbursement/gift	83.8	85.7	83.6	85.0	83.1
32. Blood inventory and forecast	82.6	78.6	83.1	88.2	79.4
**Interactive services (10)**					
33. Appointment for personal donation	43.5	82.1***	38.7	49.5	40.0
34. Appointment for group donation	39.9	82.1***	34.7	48.4*	35.0
35 Appointment for apheresis donation	24.5	64.3***	19.6	28.0	22.5
36 Rare blood type registry and donation	15.0	57.1***	9.8	16.1	14.4
37. Appointment record and donation record query	27.3	64.3***	22.7	32.3	24.4
38. Laboratory test result query	42.7	60.7*	40.4	51.6*	37.5
39 Track the blood you donated	3.6	21.4***	1.3	3.2	3.8
40 Apply/registry for volunteer	21.7	35.7	20.0	30.1*	16.9
41 On-line consultation	59.3	71.4	57.8	58.1	60.0
42 Suggestion/complains via internet or e-mail	53.0	82.1**	49.3	59.1	49.4

Levels of statistical significance for differences between provincial vs. regional and coastal vs. inland blood centers are marked with: *** (p<0.001), ** (p<0.01) or * (p<0.05).

#### Technical aspects

Websites gave a high percentage of seven items concerning technical aspects (item 3-item9; 84.2%-100.0%). Two items concerning the navigability of websites, website map (item1, 5.5%) and usable searcher engine (item2, 26.5%), were poorly presented. Only 1.6% websites offered information in one or more foreign languages (item 10).

#### Information quality

Information quality were poor, as only three items concerning mission statement (item 12), copyright (item 17) and currency of information (item 19) were found on >80% websites. The majority of websites did not provide author’s name and qualification (item 11, concerning authority of information, 18.6%) or the sources and publish date of medical information (item 12, concerning attribution of information, 13.4%). The items concerning privacy issues (item 13), transparency (item 15), financial disclosure (item 17) and legal rights and obligations (item 18) were also poorly presented.

#### Information comprehensiveness

More than 80% websites presented most of the items (item 20–23, 25, 27, 29–32) concerning information needed by website users who are considering or planning blood donation. Fewer websites presented information about rights and responsibility of blood donors (item 24, 70.0%), and less than half websites gave information about possible adverse events during blood donation (item 26, 47.4%) and pre-donation health evaluation form (item28, 46.3%).

#### Interactive services

Potential donors can make appointments and/or registrations before arriving at the blood donation site and actively retrieve information after their donations on less than half of all website (item 33–40, 3.6%-43.5%). Users can receive on-line consultation and send suggestions/complains through a little higher percentage of websites (item 41–42; 59.3% and 53.0%). Compared to regional blood centers, significantly higher percentages of provincial blood centers’ websites had these functionalities, except for apply/registry for volunteer (item40) and on-line consultation (item41).

### Website evaluation scores and the blood supply capacity of blood centers

**Fig 4:**
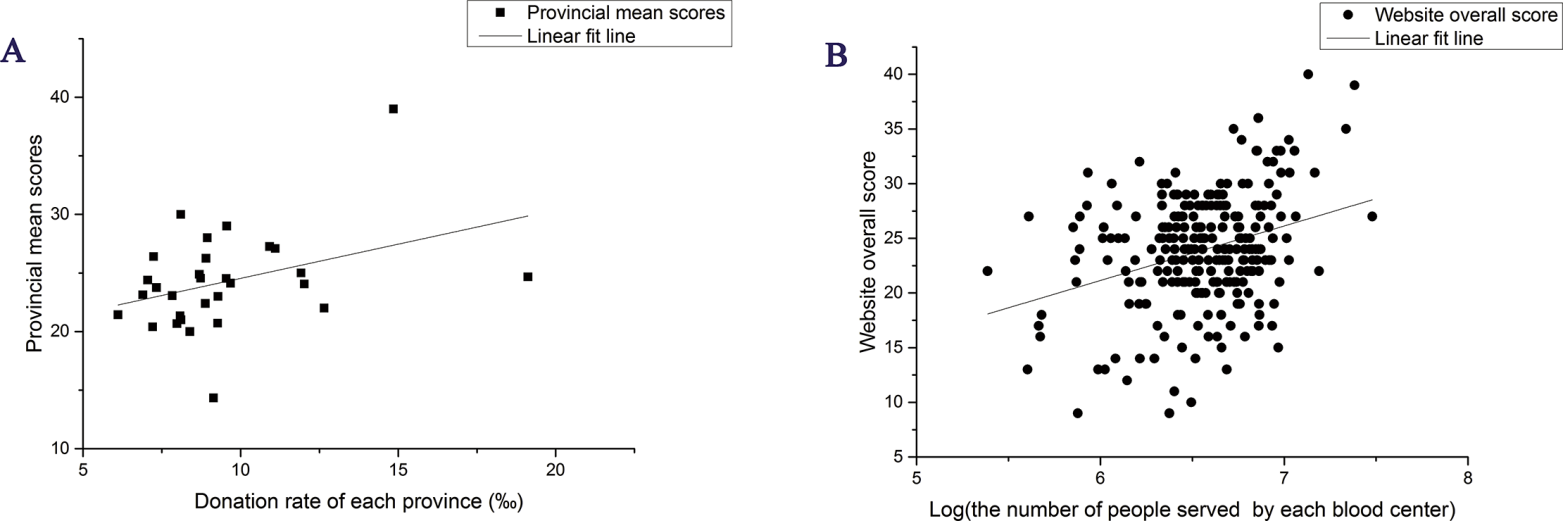
Linear regression plots. Regression plots showing the relationship between the provincial mean scores for websites and the donation rate of each province (A), and the relationship between website overall scores and the number of people served by each blood center (B). The scattered points indicate data points and the lines indicate the linear regression lines.

On the provincial level, neither the number of units of blood collected by voluntary donation nor the number of donors was significantly related to provincial mean score. The donation rate (donations made by per 1000 people), however, was positively related to the provincial mean score with marginally significance (Spearmen’s correlation coefficient ρ = 0.361, p = 0.046; linear regression: r^2^ = 0. 135, b = 0.584, p = 0.046) (Fig 4A). On the individual level, a natural log transformation was applied to the number of people served by blood center based on the scatter plot taking overall score as dependent variable and the number of people served by blood center as independent variable. The overall score of each website was also positively and significantly related to the log of number of people served by the same blood center (ρ = 0.221, p<0.001; linear regression: r^2^ = 0. 098, b = 4.989, p<0.001) (Fig 4B).

## Discussion

This study was designed to assess the quantity and quality of blood centers’ official websites in China. With the ongoing growth of clinical blood demand and rapid growth of internet users in China, providing comprehensive information and interactive services through official websites seems a useful way for acknowledging interested users and keeping in touch with peoples who had already donated blood.

The number of official websites was firstly counted and 253 blood centers had a working official website (72.3%). Although no statistical data on blood centers’ websites have been reported, this overall percentage was comparable to those of Italian public hospitals (64.3%) [15], Greek public hospitals (43.5%) [24] and clinics in the United States (75.3%) [25]. It implies that the website construction of Chinese blood centers have caught up with the average pace of healthcare facilities after the rapid development. However, using a newly developed instrument, the evaluation of websites revealed that the mean overall score of all websites was just 24.7 out of 42 and nearly three quarters of all websites were of fair quality. These results highlight the necessity to improve the quality of all blood centers’ websites.

Comparison between the four sub-scores revealed that websites could be rated as fair-good in information comprehensiveness and technical aspects, but poor in information quality and interactive services. Several technical items were not found in many websites, despite the overall fair-good sub-scores. A website map and a usable searcher engine were provided by less than 10% websites, which may influences user’s ability to find specific content, their capacity to process the content and their perceptions of experience [26]. Only four websites provide content in foreign languages, which may be linked to the generally low proportions of foreign donors in many blood centers in China. Among all the essential information for blood donor counselling, the information on possible adverse event and pre-donation health evaluation form were poorly presented. Information about adverse event during blood donation could help avoiding adverse events and elevating donor return rate. However, on the other hand, it may increase anxiety and fear of some potential donors [27], which may be the reason for not mentioning it on websites.

With regard to information quality, the poorly represented disclosure of authority, attribution of sources, financial issues and disclaimers would all have negative influence on users’ information judgments on reliability [28] and credibility (e.g., trustworthiness, expertise) of websites and ultimately the user’s decisions regarding blood donation. Blood centers keep and disclosure sensitive health information such as HIV infection status, so they should take privacy and confidentiality issues very seriously. Although China is moving to strengthen online privacy, the privacy legislation is far from complete [29] and the recognition of privacy remain obscure and deviated [30], which may contribute to the lack of clarification on privacy policies. Generally speaking, these websites passively delivery information other than interactively communicate with users and provide individualized supports and services. User-initiated appointment can reduce waiting time [31], improve blood donation experience and donor satisfaction [32]. Users can only make various types of appointments in 15.0%-43.5% websites, which may cause inconvenience and hinder the blood donation decision among potential donors.

As we have hypothesized, the administrative level and the location of blood centers both had significant impacts on the number and quality of their official websites. Blood centers with higher administrative level (i.e. provincial blood centers) and blood centers located in economically more developed region have more financial allocations and supportive resources. The effect of economic factors on the quality of websites is consistent with the results of fertility websites and obstetrics and gynecology department websites [16]. These results suggest that increasing financial funds and other related resources maybe a way for improving website quality. The evaluation scores can also reflect the actual working capacity of blood centers in some extent. The donation rate in each province was positively related to the means of overall score of all the blood centers’ websites in the same province. The number of people served by each blood center, which can be considered as an indicator of local blood demand, was also positively related to the overall score of the same blood center.

The strength of this nationwide cross-sectional study lies in the large sample of websites and the validity of evaluation instrument, in terms of inter-coder reliability. However, there are a number of limitations of this study. Firstly, there are many theoretical framework and proposed criteria for assessing a website. Our evaluation sections and items were adopted based on the responsibilities and functions of blood centers in China. Therefore some evaluation items may not be adaptable for blood centers websites form other countries and a different scale could possibly have changed the results. Secondly, as regard to specific functionalities such as online appointment, it is not practical to get local donors’ information to fully assess them on every website. Raters examined the presence of specific pages or tables for these functionalities, and tried to type information into corresponding tables or click specific buttons without the final information submission. Thus possible malfunction of these services may be overlooked, causing overrating of the interactive services. Thirdly, blood centers in China nowadays are utilizing cutting-edge technologies including social media applications like Weibo and Wechat to promote blood donation. Due to the technical differences between these mobile applications and websites, our instrument was not suitable to evaluate these useful applications. Therefore, our study cannot comprehensively depict all the effort in online communication and services made by blood centers in China.

## Conclusions

This is the first study to develop a website evaluation instrument specifically for websites of blood centers, and can be considered a baseline health informatics survey in the field of transfusion medicine. A high proportion of blood centers in China provided an official website, but the majority of websites were of fair quality. Especially, only a few websites provided information and services likely to increase the credibility of blood centers and facilitate blood donation. These findings can be used to guide the future improvement of blood centers’ websites.

## Supporting information

S1 Fig Regional division of China based on economics. This figure was modified from the Wikipedia Commons. CC BY-SA 3.0, https://commons.wikimedia.org/wiki/File:China_edcp_location_map.svg.
(TIF)

S1 Table Division of provinces based on the level of economic development. (XLSX)

S2 Table The number of people served by each blood center. (XLSX)

S3 Table The donation data for each province. (XLSX)

S4 Table Detailed evaluation score for each website. (XLSX)
