# Supplementary figures and images for: The official websites of blood centers in China: A nationwide cross-sectional study
Source: PLoS One. 2017 Aug 9;12(8):e0182748. doi: 10.1371/journal.pone.0182748 (PMC5549989; doi:10.1371/journal.pone.0182748)

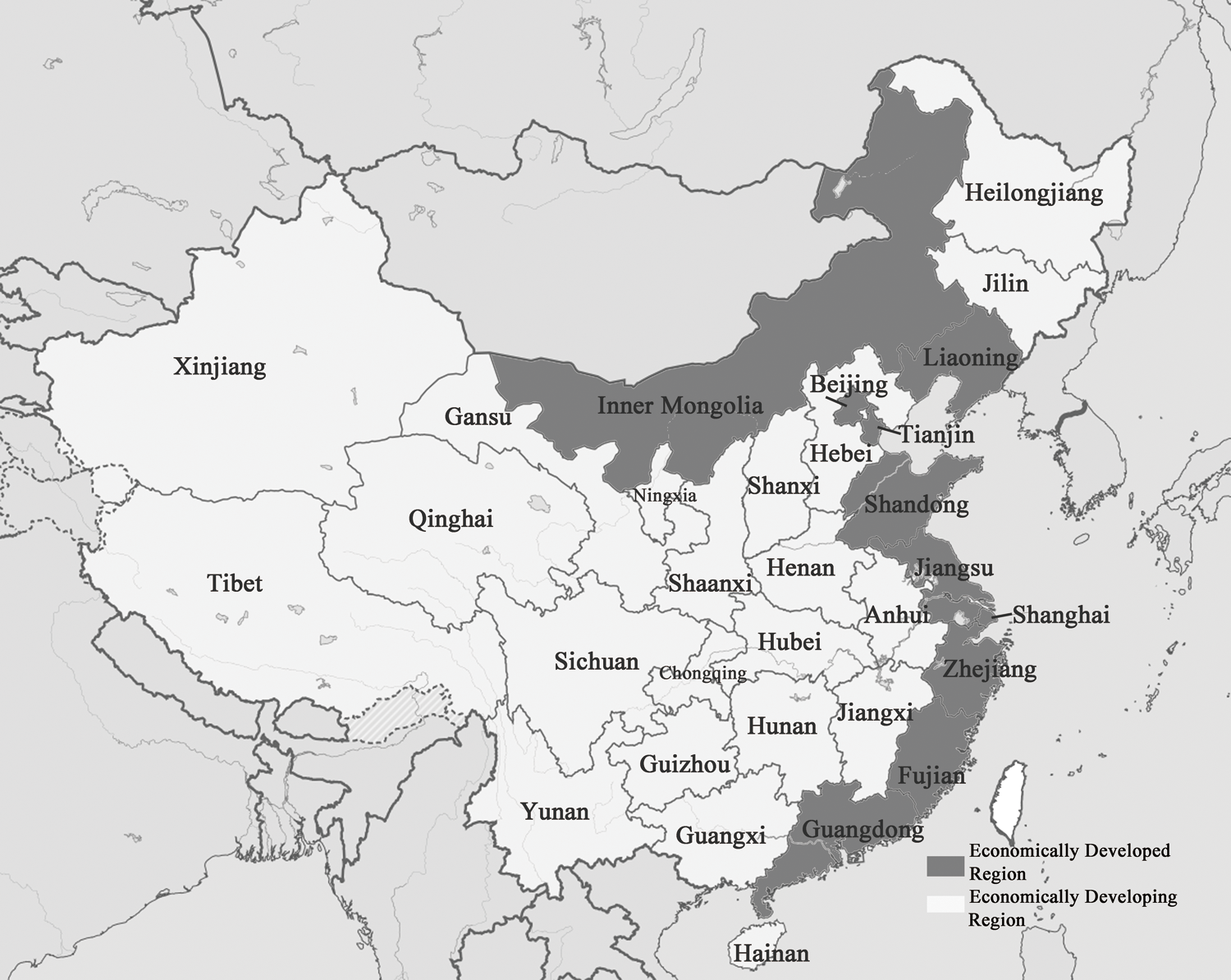

Supplement: S1 Fig — This figure was modified from the Wikipedia Commons. CC BY-SA 3.0, https://commons.wikimedia.org/wiki/File:China_edcp_location_map.svg. (TIF) [file pone.0182748.s001.tif]
